# Modulation of gene transcription and epigenetics of colon carcinoma cells by bacterial membrane vesicles

**DOI:** 10.1038/s41598-018-25308-9

**Published:** 2018-05-09

**Authors:** Svitlana Vdovikova, Siv Gilfillan, Shixiong Wang, Mitesh Dongre, Sun Nyunt Wai, Antoni Hurtado

**Affiliations:** 1 Department of Molecular Biology, Umeå University, 90187 Umea, Sweden; 2 The Laboratory for Molecular Infection Medicine Sweden (MIMS), Umeå University, 90187 Umea, Sweden; 3 Umeå Centre for Microbial Research (UCMR), Umeå University, 90187 Umea, Sweden; 4 Breast Cancer Research group, Nordic EMBL Partnership, Centre for Molecular Medicine Norway (NCMM), University of Oslo, 0318 Oslo, Norway

## Abstract

Interactions between bacteria and colon cancer cells influence the transcription of the host cell. Yet is it undetermined whether the bacteria itself or the communication between the host and bacteria is responsible for the genomic changes in the eukaryotic cell. Now, we have investigated the genomic and epigenetic consequences of co-culturing colorectal carcinoma cells with membrane vesicles from pathogenic bacteria *Vibrio cholerae* and non-pathogenic commensal bacteria *Escherichia coli*. Our study reveals that membrane vesicles from pathogenic and commensal bacteria have a global impact on the gene expression of colon-carcinoma cells. The changes in gene expression correlate positively with both epigenetic changes and chromatin accessibility of promoters at transcription start sites of genes induced by both types of membrane vesicles. Moreover, we have demonstrated that membrane vesicles obtained only from *V. cholerae* induced the expression of genes associated with epithelial cell differentiation. Altogether, our study suggests that the observed genomic changes in host cells might be due to specific components of membrane vesicles and do not require communication by direct contact with the bacteria.

## Introduction

During growth, both Gram-negative and Gram-positive bacteria release membrane vesicles (MVs) from the bacterial surface. MVs secretion can occur in different environments including aquatic environments, during formation of biofilms and in the infected host^1^. They are spherical membranous particles with 20–300 nm diameters, and through their formation may entrap common or specific bacterial components such us periplasmic components, lipopolysaccharides (LPS), peptidoglycan, phospholipids, nucleic acids, proteins, ion metabolites, enzymes, and specific bacterial components^2–4^. These MVs need to be considered in many contexts of bacterial interactions with the host environment, where they may be involved in extracellular signalling. Moreover, MVs serve as long distance vehicles of multifunctional bacterial cargos including several toxins and immune modulators^5–17^. MVs may use several different pathways to internalize into the host cells, including endocytosis as well as fusion with the eukaryotic plasma membrane^18^. Previously, we reported that the cytolethal distending toxin (CDT), a genotoxin associated with MVs from *Aggregatibacter actinomycetemcomitans*, was internalized in both HeLa cells and human gingival fibroblasts (HGF) via a mechanism of MVs fusion with lipid rafts in the plasma membrane^10^. The exact intracellular fate of MVs and how they interact with intracellular organelles still remains unclear. MVs-associated factors may cause very distinct and biologically relevant effects in the target cells such as: (1) empowering bacteria to subvert both the host immune and microbiome-associated defence systems, (2) preventing disease development or (3) mediating the anti-inflammatory and intestinal barrier protection^5,19–21^.

Epigenetic modifications in eukaryotes contribute to modulate gene transcription and they may include DNA methylation, histone modifications and modulation of long non-coding RNA and microRNA expression^22^. DNA methylation is mainly associated with transcriptional gene repression, whereas histone modifications are correlated with either transcriptional gene activation or repression. Moreover, the state of chromatin compaction affects gene transcription. Previous studies have shown that bacterial pathogens are able to modulate the host transcriptional profile through epigenetic modifications. For instance, changes in DNA methylation and histone acetylation of host cells have been identified in response to infections of several types of bacteria such as *Helicobacter pylori*^23,24^, uropathogenic *Escherichia coli*^25^, *Porphyromonas gingivalis*^24^, *Fusobacterium nucleatum*^26^, *Listeria monocytogenes*^24,27,28^, *Legionella pneumophila*^29^ or commensal *Bacteroides vulgatus*^30^. Interestingly, some studies have identified that the intestinal microbiome alters the miRNA expression of the gut as a mechanism to maintain the intestinal symbiotic system^31–34^. Moreover, short-chain fatty acids produced by intestinal microbiota are known to act as a histone deacetylase inhibitors^35^, which suggests that commensal bacteria might also induce epigenetic changes into the gut microbiota. However, the mechanism by which bacteria might induce epigenetic changes of the colon tissue is not fully elucidated. Previously, it was proposed that commensal bacteria regulate intestinal inflammation through DNA methylation of the TLR4 gene^36^. Furthermore, an epidemiological study suggested that an oral bacterium, *Fusobacterium nucleatum*, might contribute to epigenetic changes in colon carcinoma tissue^37^. Yet, it is not well understood how the communication between bacteria and host occurs, or whether any released component from bacteria might be responsible of such genomic effects. To our knowledge, it is unknown whether bacterial MVs can also target host cell epigenetics as an alternative to the presence of whole bacteria. In this study, we aimed to investigate the genomic and epigenetic consequences of co-culturing HCT8 colorectal carcinoma cells with MVs isolated from pathogenic and non-pathogenic commensal bacterial strains, *Vibrio cholerae* strain C6706 and *Escherichia coli* K-12 strain MC1061, respectively.

## Results

**Figure 1:**
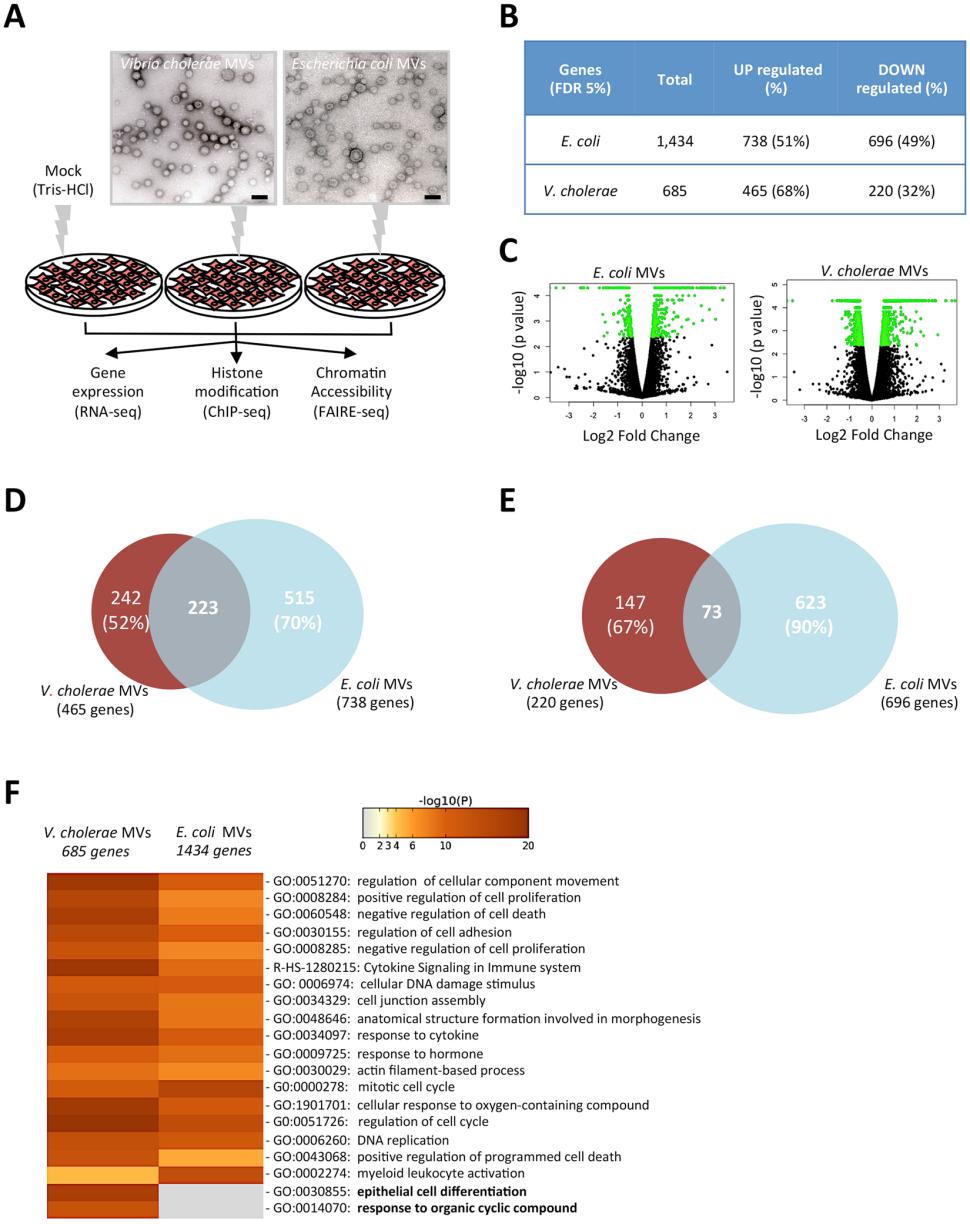
Bacterial membrane vesicles (MVs) target the gene transcription of HCT8 colorectal carcinoma cell line. (**A**) HCT8 cells were co-cultured with MVs from *E. coli*, *V. cholerae* or mock-treated (control) for 5 hours. The setup of the study with the following methods is shown: RNA sequencing, ChIP sequencing (H3K4me3) and FAIRE sequencing (nucleosome-free DNA), Bars; 200 nm. (**B**) Table of RNA transcripts significantly regulated by MVs from *E. coli* or *V. cholerae* compared to mock. (**C**) Volcano plots of differentially regulated host cell genes by MVs from *E. coli* or *V. cholerae* compared to mock. (**D**) Venn Diagram showing the overlap of upregulated genes between HCT8 cells co-cultured with MVs from *E. coli* and *V. cholerae*. (**E**) Venn Diagram showing the overlap of downregulated genes between HCT8 cells co-cultured with MVs from *E. coli* and *V. cholerae*. (**F**) Gene ontology enriched terms for genes differentially regulated in HCT8 cells co-cultured with MVs from *E. coli* or *V. cholerae*.

In this study we examined the impact of MVs isolated from *V. cholerae* and *E. coli* on HCT8 cells from human ileocecal colorectal adenocarcinoma. MVs were isolated from the supernatants of bacteria grown to late-stationary phase and visualised with Transmission Electron Microscopy (Fig. 1A, upper panel). Then, HCT8 cells were co-cultured with MVs from *E. coli* or *V. cholerae* or mock-treated for 5 hours. After incubation, cells were collected to further determine changes in gene expression (by RNA sequencing), histone mark modification of active promoters (by H3K4me3 ChIP sequencing) and chromatin accessibility (by FAIRE sequencing) (Fig. 1A, lower panel).

### MVs isolated from *E. coli* and *V. cholerae* affect the gene expression of HCT8 cells

To determine the effect of MVs on colon carcinoma cells, we first assessed the influence of *E. coli* MVs and *V. cholerae* MVs on global gene expression in HCT8 cells. In order to identify the primary impact of both MVs on host cell gene expression, we used an early incubation time point of 5 hours. To determine differential regulation of transcripts by specific bacterial MVs we isolated RNA from MVs-treated cells and performed RNA sequencing (Fig. 1A). We identified a total of 1,434 and 685 genes differentially regulated by *E. coli* MVs and *V. cholerae* MVs, respectively (Fig. 1B). We considered 2-fold changes in gene expression as differential regulation when cells treated with MVs were compared to untreated cells. Around 51% (738 out of 1,434) of the genes affected by *E. coli* MVs were significantly upregulated at least two-fold when compared to control cells (Fig. 1B and left panel of Fig. 1C). Moreover, we observed that around 68% (465 out of 685) of the genes affected by *V. cholerae* MVs treatment were significantly upregulated at least two-fold when compared to control cells (Fig. 1B and right panel of Fig. 1C). The comparison of gene transcripts significantly upregulated by both types of MVs revealed a substantial overlap and included 223 genes (Fig. 1D). Furthermore, the analysis of the downregulated genes revealed a more limited overlap (73 genes) between those affected by *V. cholerae* and *E. coli* MVs (Fig. 1E). By contrast, we identified that around 70% of the upregulated and 90% of the downregulated genes affected by *E. coli* MVs were not significantly affected by *V. cholerae* MVs, whereas only 52% of the upregulated and 67% of the downregulated genes affected by *V. cholerae* MVs were not significantly affected by *E. coli* MVs (Fig. 1D,E). The results of this analysis indicated that the MVs of the two species of bacteria induced differential gene expression in HCT8 cells. However, when we performed quantification of the global expression of genes regulated by treatment of cells with either of the two species of MVs we identified less differential gene expression. The analysis indicated that the significantly upregulated genes in the cells treated with *E. coli* MVs could also be upregulated by *V. cholerae* MVs and vice versa. The quantification of the global expression indicated that genes significantly downregulated by *E. coli* MVs were not downregulated by *V. cholerae* MVs and vice versa. (Supplementary Fig. S1). Altogether, these results suggested that both *E. coli* and *V. cholerae* MVs could stimulate a similar set of gene transcripts. Furthermore, we investigated how the genes regulated by both MVs relate to cell-specific functions. We performed pathway analysis with the distinct subset of genes modulated by *E. coli* MVs or *V. cholerae* MVs treatment (1,434 and 685 genes, respectively). The results revealed that the genes regulated by both types of MVs showed enrichment of common GO terms. Moreover, the GO term analysis revealed epithelial cell differentiation and response to organic cyclic compound as GO terms specifically enriched for treatment with *V. cholerae* MVs (Fig. 1F). The changes in gene expression were confirmed by realtime PCR after exposing HCT8 cells to different concentrations of MVs. We identified that gene transcripts enriched at GO term epithelial cell differentiation were exclusively regulated by *V. cholerae* MVs (Supplementary Fig. 2A). Moreover, the expression of gene transcripts commonly upregulated by both MVs was validated by realtime PCR (Supplementary Fig. 2B). Furthermore, we aimed to validate whether the selective role of the pathogenic MVs might be observed in other cancer cell lines. Hence, we exposed MCF-7 breast cancer cells to different concentrations of MVs from *V. cholerae* and determined the expression of genes enriched towards epithelial cell differentiation (Supplementary Fig. 2C). The exposure of breast cancer cells to different concentrations of MVs from *V. cholerae* did not increase the expression of the cell differentiation genes investigated as was observed in colon cancer cells, which suggested a tissue-specific role of the pathogenic bacteria.

**Figure 2:**
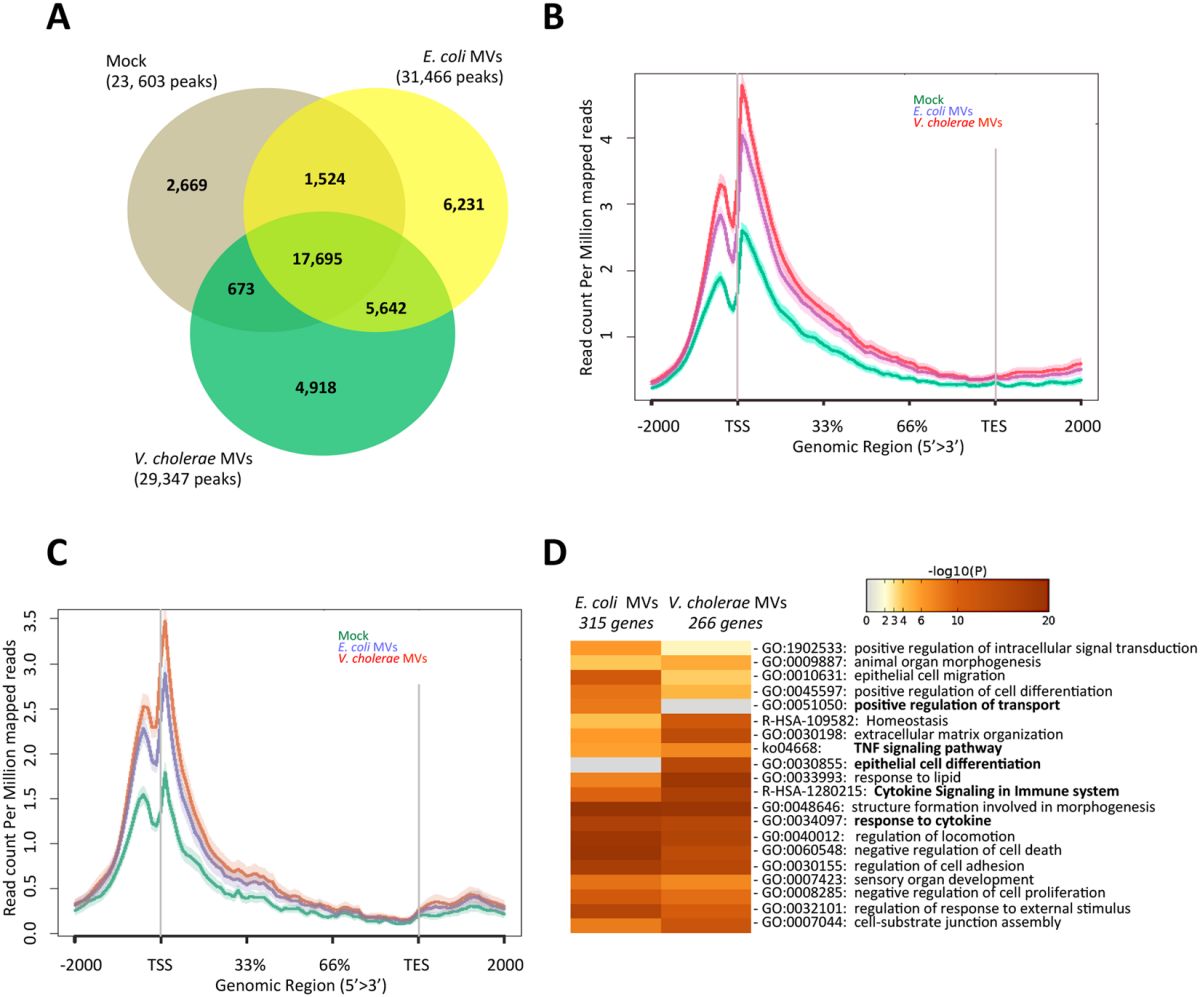
MVs influence the epigenetic mark H3K4me3 at promoters and TSS of gene transcripts in HCT8 colorectal cancer cell line. (**A**) Venn Diagram showing the overlap of H3K4me3 peaks of HCT8 cells treated with MVs from *E. coli*, *V. cholerae* or mock-treated cells. (**B**) H3K4me3 signal at transcription start sites (TSS) of upregulated genes from HCT8 cells co-cultured with *E. coli* MVs (738 genes). (**C**) H3K4me3 signal at TSS of upregulated genes from HCT8 cells co-cultured with *V. cholerae* MVs (465 genes). (**D**) Gene ontology enriched terms for upregulated genes and H3K4me3 binding in HCT8 cells co-cultured with MVs from *E. coli* or *V. cholerae*.

To determine whether MVs have an impact on the epigenetics of colon carcinoma cells, we assessed the H3K4me3 interaction at genome-wide level by ChIP sequencing. H3K4me3 is a histone mark modification at the transcription start sites (TSS) that correlates positively with active transcription. We performed H3K4me3 ChIP sequencing of the HCT8 cells, and their binding interactions were called using MACS^38^, where mock and MVs-treated HCT8 cells were analysed. We found 31,466 H3K4me3 binding events with *E. coli* MVs treatment, 29,347 with *V. cholerae* MVs treatment and 23,603 with mock treatment (Fig. 2A). The comparison of binding events revealed a substantial overlap in H3K4me3 binding sites among the three conditions (75% for mock: 17,695 out of 23,603; 56% for *E. coli* MVs: 17,695 out of 31,466; and 60% for *V. cholerae* MVs: 17,695 out of 29,347). Subsequently, we investigated whether the upregulation of gene transcripts, due to the co-culture with MVs from *E. coli* or *V. cholerae*, correlated with an increase of histone mark H3K4me3 at their TSS. Hence, we analysed the distribution of reads from H3K4me3 Chip-sequencing experiments in a window covering the transcription start sites (TSS) and the termination end sites (TES) at genes that were upregulated after MV treatment. We observed that both pathogenic and non-pathogenic MVs increased the signal with H3K4me3 around the TSS of genes (relative to control treated cells) that were upregulated by *E. coli* MVs (Fig. 2B). In the same regard, we identified in both types of MVs an increase of H3K4me3 signal around the TSS of genes upregulated by *V. cholerae* MVs (Fig. 2C). These results were in agreement with the expression data (Fig. 1) and supported that the MVs from both pathogenic and non-pathogenic bacteria have a positive impact on the activation of gene transcripts in HCT8 cells. Next, we performed a pathway analysis within the MVs-regulated genes associated with H3K4me3 peaks. This analysis showed enrichment of common GO terms. Moreover, the analysis revealed a specific gene signature associated with positive regulation of transport in case of *E. coli* MVs treatment and epithelial cell differentiation in case of *V. cholerae* MVs treatment (Fig. 2D), which confirmed our results from Fig. 1D. All together, these results supported the idea that MVs from pathogenic *V. cholerae* might have an impact on the set of genes involved in differentiation of colon carcinoma cells.

**Figure 3:**
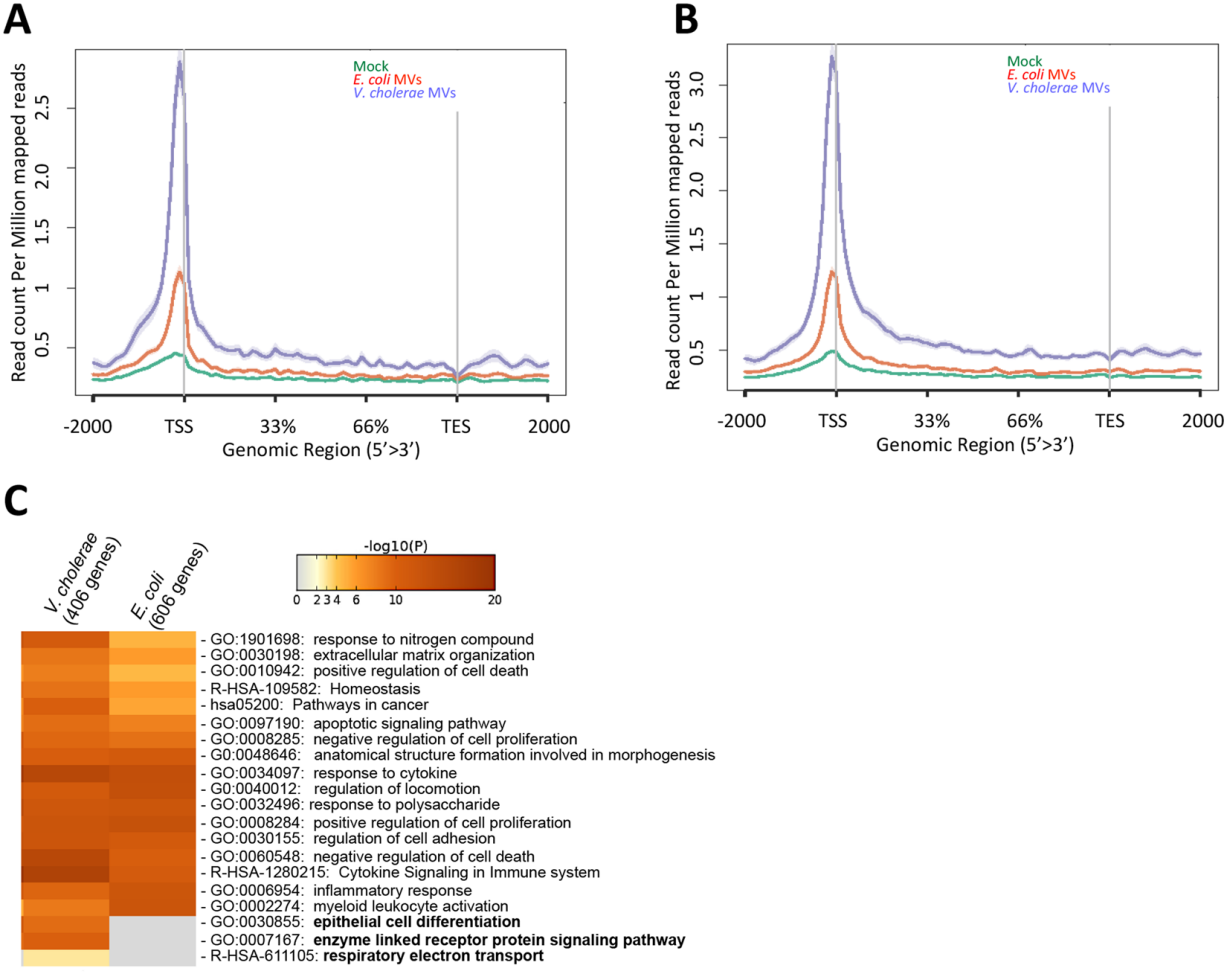
Influence of MVs on the opening of chromatin at promoters and TSS of upregulated gene transcripts in HCT8 colorectal cancer cell line. (**A**) FAIRE signal (nucleosome free-DNA) at TSS of upregulated genes (465 genes) in HCT8 cells co-cultured with MVs from *V. cholerae*. (**B**) FAIRE signal at TSS of upregulated genes (738 genes) in HCT8 cells co-cultured with MVs from *E. coli*. (**C**) GO enriched terms of genes upregulated and with FAIRE signal in HCT8 cells co-cultured with MVs from *E. coli* or *V. cholerae*.

Previously, it has been reported that transcriptional activation correlates with an increase of nucleosome-free DNA^39^, which is an event that facilitates the binding of basic transcriptional machinery and activity of RNA polymerase. Therefore, we aimed to investigate whether MVs might influence the DNA-accessibility at the TSS of MVs-targeted genes identified at Fig. 1. We performed formaldehyde-assisted isolation of regulatory elements (FAIRE) coupled with high-throughput sequencing to identify euchromatic regions of the genome^40^. FAIRE is a method used for determining open chromatin regions (nucleosome-free DNA) that are accessible to transcription factors and the basal transcriptional machinery. The DNA accessibility of a promoter or TSS correlates with active gene transcription. Hence, HCT8 cells were treated with *E. coli* MVs, with *V. cholerae* MVs or mock-treated for 5 h, as indicated in Fig. 1A. Furthermore, nucleosome-free DNA was isolated and sequenced. Finally, we examined the FAIRE signal (nucleosome-free DNA) at genes upregulated by MVs from *V. cholerae* (Fig. 3A) and by MVs from *E. coli* (Fig. 3B). MVs from both bacterial species were able to open the chromatin at TSS of genes leading to upregulation of target genes. Interestingly, the effect of *V. cholerae* MVs on the chromatin at TSS was more pronounced compared to that of *E. coli* MVs. Next, we performed pathway analysis of upregulated genes that contained FAIRE signal. The results revealed that the upregulated genes from both types of bacterial MVs were significantly enriched for the majority of the terms. Interestingly, this analysis revealed that a few pathways were exclusively enriched for *V. cholerae* MVs (Fig. 3C). Among others, the term of epithelial cell differentiation was one of the most significant pathways identified, which confirmed additionally our findings with the histone mark H3K4me3 (from Fig. 2D).

### Differential effects of MVs isolated from pathogenic and non-pathogenic bacteria on the target gene**s**

**Figure 4:**
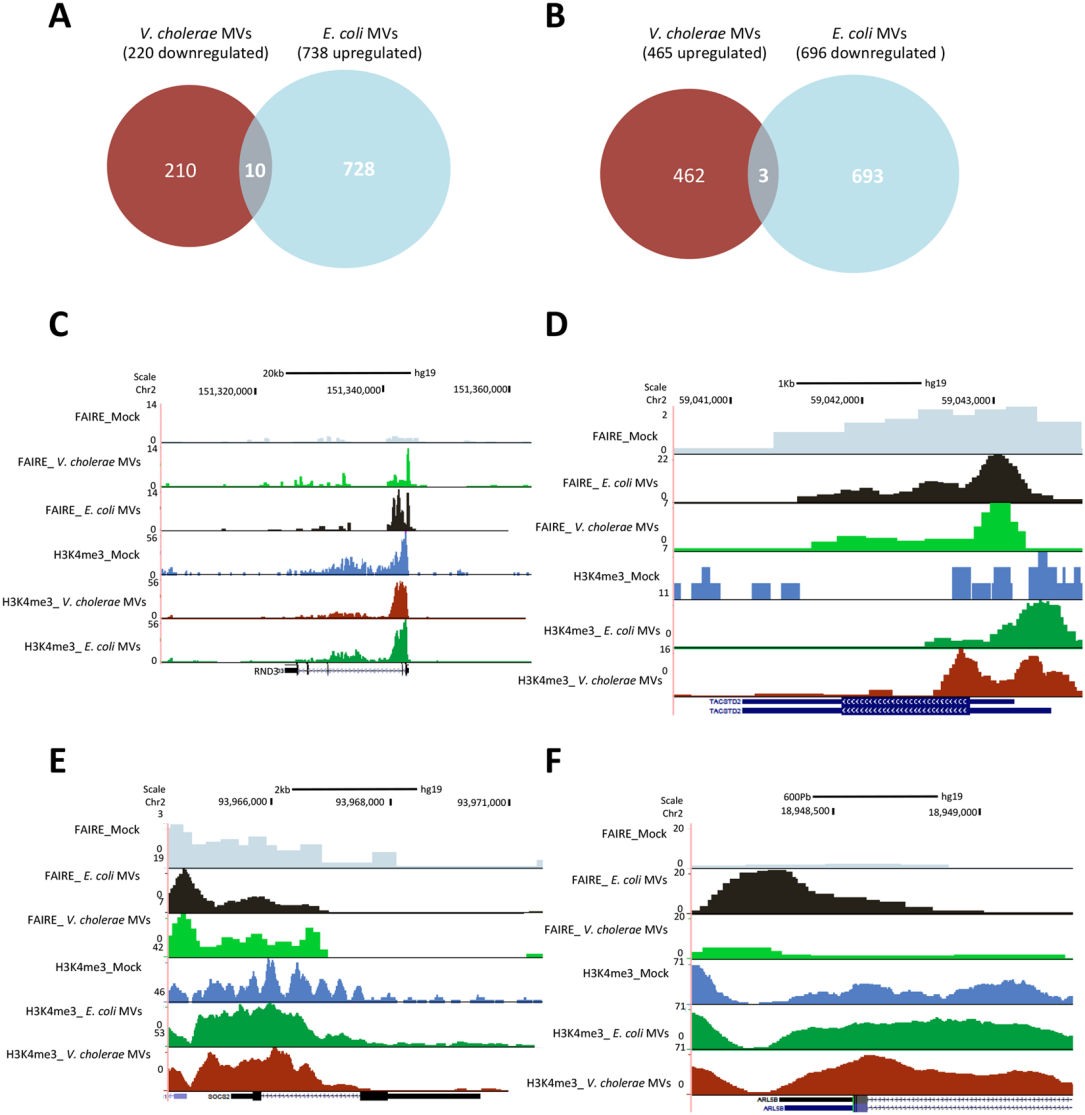
Influence of MVs on differentially regulated gene transcripts in HCT8 colorectal cancer cell line. (**A**) Venn Diagram showing the overlap of genes upregulated by *E. coli* MVs and downregulated by *V. cholerae* MVs. (**B**) Venn Diagram showing the overlap of genes downregulated by *E. coli* MVs and upregulated by *V. cholerae* MVs. (**C**–**F**) Examples of gene transcripts regulated in opposite way: (**C**) RND3 (*E. coli* up and *V. cholerae* down), (**D**) TACSTD2 (*E. coli* up and *V. cholerae* down), (**E**) SOCS2 (*E. coli* up and *V. cholerae* down) and (**F**) ARL5B (*E. coli* down and *V. cholerae* up).

Our results suggested that a high number of gene transcripts, significantly regulated in colorectal carcinoma cell line, were commonly induced after both MV treatments. Interestingly, a subset of genes associated with epithelial cell differentiation were consistently upregulated exclusively by *V. cholerae* MVs. Next, we aimed to determine whether *V. cholerae* MVs could differentially regulate the gene transcription compared to *E. coli* MVs and vice versa. For this analysis, we compared the gene transcripts upregulated by *E. coli* MVs with the gene transcripts downregulated by *V. cholerae* MVs (Fig. 4A). The results revealed that a very low number of genes upregulated by *E. coli* vesicles were in fact downregulated by *V. cholerae* vesicles (1.3%, 10 out of 738 genes). Moreover, we compared the gene transcripts upregulated by *V. cholerae* MVs with the gene transcripts downregulated by *E. coli* MVs (Fig. 4B). We also observed that a very low number of genes upregulated by *V. cholerae* MVs were in fact downregulated by *E. coli* MVs (0,6%, 3 out of 465 genes). Despite the fact that a few of these genes are differentially regulated by *V. cholerae* MVs and *E. coli* MVs, we could identify some genes, which are important in the development of colon cancer. For instance, RND3 (Fig. 4C), TACSTD2 (Fig. 4D) and SOCS2 (Fig. 4E) were examples of genes upregulated by *E. coli* MVs and downregulated by *V. cholerae* MVs. Interestingly, *V. cholerae* MVs increased the chromatin accessibility at the TSS of the target genes, whereas the *E. coli* MVs did not have such effect (Fig. 4C–E). Finally, we also identified the gene ARL5b (Fig. 4F) to be upregulated by *V. cholerae* MVs and downregulated by *E. coli* MVs. In agreement with the gene expression data, an increase of the histone mark H3K4me3 signal was observed at the TSS of ARL5b gene in cells treated with the *V. cholerae* MVs when compared to *E. coli* MVs treatment or mock (Fig. 4F).

## Discussion

This study has demonstrated that MVs from pathogenic and commensal bacteria have a significant and global impact on the gene expression of colon carcinoma cells. Interestingly, the co-culture of HCT8 cells with both pathogenic and non-pathogenic MVs increased significantly the transcription of common targets genes, which suggests a nonspecific effect of MVs. Importantly, the changes in gene expression in our study correlated with both epigenetic changes and chromatin accessibility of promoters and TSS of genes induced by both pathogenic and non-pathogenic MVs. The changes in gene expression observed when bacteria are present might be due to any of the components of MVs rather than to direct contact between bacteria and the host cell, as suggested previously^25,27^. The release of MVs is a commonly occurring process when bacteria adapt to different environments both *in vivo* and *in vitro*. For example, *Salmonella Typhimurium* releases MVs during its intracellular growth in epithelial cells and macrophages^41^. Moreover, MVs produced by *Helicobacter pylori*, a causal organism of gastric ulcers, release a virulence factor inside the gastric cells^42^. Another example with pathogenic bacteria is related with *Neisseria meningitides*, which has been demonstrated to release MVs containing the endotoxin LPS in blood^43^.

Mechanistically, most viral or bacterial infections induce host DNA methylation indirectly via chronic inflammation, however recent studies have indicated that some viruses have direct epigenetic effects at gene transcripts of host cells^23,44–46^. Our study reveals that genes enriched with the GO term cellular immunity were identified among the genes commonly upregulated and enriched with H3K4me3 signals by both MVs in colon cancer cells. This supports that MVs might directly control the transcription of the host cells and probably their response to the immune system. However, the bacterial components that contribute to the increase of transcription in the host cell and their interplay with the immune function are important questions still unresolved. Previously, it was reported that bacterial LPS cause inflammation by means of activating p38 MAPK pathway and the nuclear transcription factor–κB (NF-κB)^26,47,48^. Furthermore, chronic inflammation causes epigenetic modifications leading to carcinogenesis, as described in the case of *H. pylori*^49–51^. In response, the host cell activates the TGF-β1 pathway to counteract the bacteria-induced NF-κB recruitment to the Il-6 promoter, which causes a reduction of histone acetylation/phosphorylation of the promoter^30,48^.

Our study also demonstrates that MVs from pathogenic bacteria *V. cholerae* have a selective impact on gene transcripts associated with epithelial cellular differentiation in colon cancer cells. Interestingly, MVs from *V. cholerae* increased the expression of genes that have a key role in cellular differentiation. For instance, the expression of the nuclear receptor for Vitamin D (VDR) genes were selectively upregulated by MVs from *V. cholerae* in colon carcinoma cells. In fact, the expression and the activation of VDR promotes the differentiation of colon carcinoma cells^52^. Hence, the increased expression of VDR might induce the expression of genes that promote differentiation and the formation of adherens junctions (AJ). The core complex of AJ is formed of transmembrane cell-cell adhesion molecules cadherins and adaptor proteins. Clustering of these molecules at junctions regulates cellular responses, with crucial effects on the physiology and on the epithelial cell differentiation^53^. Our study also reveals that MVs from *V. cholerae* induces the expression TJP1 (also know how ZO-1) and its protein product is a component of the adherens junctions. Interestingly, the decrease in ZO-1 expression reduces human trophoblast cell-cell fusion and differentiation^54^. Altogether, our results support the hypothesis that components specific to *V. cholerae* MVs would be responsible for the regulation of gene transcripts related to the differentiation of colon carcinoma cells. Interestingly, it has been reported that the major virulence factor of *V. cholerae*, cholera toxin (CT), suppresses carcinogenesis in a mouse model of inflammation-driven sporadic colon cancer^55^. Now, our study adds a value to the emerging notion that MVs produced by *V. cholerae* might impact the transcription of selective genes and, therefore, might play a role in colon cancer differentiation. Recent studies have also shown that protease secreted by *V. cholerae* induced apoptosis in breast cancer cells by ROS-mediated intrinsic pathway and inhibited tumour growth in mice model^56,57^. Bacterial pathogens can exploit several eukaryotic signalling pathways during an infection to disrupt host-signalling pathways for bacterial survival and replication. They have evolved specific effector proteins to hijack host cell signalling, including MAPK signalling, G-protein signalling, signals controlling cytoskeletal dynamics, innate immune responses, and epigenetic modifications for their own benefit^58^. Future studies should aim to identify the specific MVs-associated factors from *V. cholerae* responsible for modulation of host cell selective gene expression. Another challenge is to understand how these factors work together to orchestrate a successful infection by bacterial pathogens. Finally, the results of this study also reveal that the impact of the pathogenic MVs might be tissue specific. Whereas in this study we have investigated a human ileocecal adenocarcinoma cell line, the results might in the future be validated in additional colon-rectal carcinoma cells.

## Materials and Methods

### Bacterial strains, growth conditions and membrane vesicles isolation

Bacterial strains used in this study were: *E. coli* K-12 strain MC1061^17^ and *V. cholerae* C6706 strain (O1 El Tor, Inaba, Sm^R^)^9^. Strains were grown at 37 °C in either Luria–Bertani (LB) broth or on LB agar for 16 hours. MVs were isolated from bacterial culture supernatants, as described earlier^16,17^. Briefly, cultures were centrifuged and filtered to remove bacteria. Further, supernatants were ultracentrifuged at 100,000 × *g* for 2 h at 4 °C, pellets were washed and re-suspended in 20 mM Tris-HCl. The MVs concentration was estimated using the Bicinchoninic Acid (BCA) Assay kit (Thermo Scientific Pierce, Rockford, IL)^5^.

### Transmission electron microscopy (TEM)

Negative staining of isolated MVs was performed as described earlier^16^.

### Cell culture and infection conditions

Human ileocecal colorectal adenocarcinoma (HCT8) and breast adenocarcinoma (MCF-7) cells were cultured in RPMI 1640 (GIBCO) and DMEM medium respectively, supplemented with 10% heat-inactivated fetal bovine serum and penicillin/streptomycin at 37 °C in a humid 5% CO_2_ atmosphere. Cells were seeded 16 h prior the infection, medium was changed and MVs or control (20 mM Tris-HCl) were incubated with HCT8 cells (600 μg MVs per 10^6^ HCT8 cells) for 5 hours. Cells were collected for further RNA or chromatin isolation.

### Chromatin Immunoprecipitation (ChIP)

Targeted genomic regions were identified by using the cross-linking (X)-ChIP protocol as described previously^40^. After the MV infection, cells were fixed 10 min with 1% formaldehyde and then quenched with glycine (125 mM). Chromatin was incubated overnight at 4 °C with Chip grade H3K4me3 antibody (5 μg, ab8580, Abcam) and equal amounts of Protein A&G Agarose Beads (Life technologies). Library preparation for sequencing was done following the instructions of TruSeq DNA sample preparation kit from Illumina or MicroPlex Library preparation kit from Diagenode.

### Formaldehyde-Assisted Isolation of Regulatory Elements (FAIRE)

After the MV infection, cells were fixed 10 min with 1% formaldehyde and then quenched with glycine (125 mM). FAIRE experiment was performed as previously described^40^. DNA was fragmented using Bioruptor sonicator (Diagenode). Non-chromatinized DNA was isolated by phenol-chloroform extraction followed by reverse cross-link at 65 °C overnight. Purified DNA fragments were processed with MicroPlex Library preparation kit from Diagenode.

### ChIP and FAIRE sequencing data Analyses

Reads generated by the genome analyzer were aligned against the human genome using Bowtie 2 software (http://bowtie-bio.sourceforge.net/bowtie2/index.shtml) with default parameters. Peak calling was performed by MACS^38^ and HOMER version 4.7.2.

### RNA isolation and quality control

After the infection, HCT8 cells in triplicates were used for RNA extraction. Total RNA was isolated with the total RNA isolation kit according to the manufacturer’s protocol (RNeasy Mini Kit, Qiagen). NanoDrop 2000 assessed RNA yield. Sequencing libraries were prepared from 1 μg total RNA using the TruSeq stranded mRNA library preparation kit (Illumina Inc) including poly-A selection and sequenced at the SNP&SEQ Technology Platform (Uppsala) in HiSeq2500 rapid mode.

The RNAseq analysis was done by the National Bioinformatics Infrastructure Sweden (http://www.scilifelab.se/platforms/bioinformatics/, www.nbis.se). Reads were aligned to the transcriptome (hg19, UCSC database by Illumina) using Tophat2. Assembly of transcripts and differential gene expression analysis were performed using Cufflinks/Cutdiff.

### Pathway analysis

Functional interpretation of differentially expressed genes and their association to the host cell pathways for each of the MVs treatments were done using Metascape software. For each treatment, the affected genes were analyzed separately for up- and downregulated genes. We identified canonical pathways that were enriched or overrepresented, with the significance of the association between the signature and the canonical pathway measured in two ways: (1) A ratio of the number of genes from the signature that map to the pathway divided by the total number of genes that map to the canonical pathway calculated; (2) A right-sided Fisher’s exact test was used to calculate a p-value determining the probability that the association between the genes in the dataset and the canonical pathway is explained by chance alone.

## Electronic supplementary material

Supplementary figures
