# Supplementary material for: Modulation of gene transcription and epigenetics of colon carcinoma cells by bacterial membrane vesicles
Source: Sci Rep. 2018 May 9;8:7434. doi: 10.1038/s41598-018-25308-9 (PMC5943334; doi:10.1038/s41598-018-25308-9)
Supplement: Supplementary file 1 — Supplementary figures [file 41598_2018_25308_MOESM1_ESM.pdf]

Supplementary Figures

Modulation of gene transcription and epigenetics of colon carcinoma cells by bacterial membrane vesicles

Svitlana Vdovikova[1,2,3*], Siv Gilfillan[4*], Shixiong Wang[4], Mitesh Dongre[1,2,3], Sun Nyunt Wai[1,2,3#], Antoni Hurtado[4#]

Supplementary Figure 1

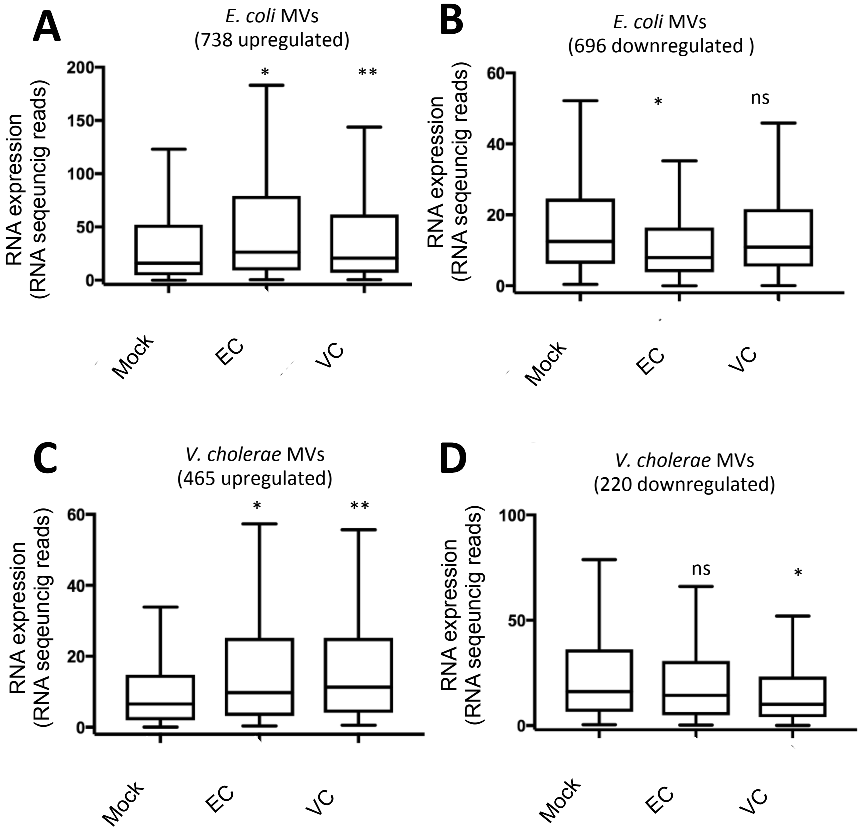

Mann–Whitney U test:
*: MVs *E. coli* (EC) vs mock ($p<10^{-6}$)
**: MVs *V. cholerae* (VC) vs mock ($p<10^{-6}$)
ns: not significant

Figure S1. Box-plot indicating the expression of differentially regulated gene transcripts of HCT8 colorectal cancer cell line. A. Up-regulated genes after *E. coli* MV treatment. **B.** Down-regulated genes after *E. coli* MV treatment. **C.** Up-regulated genes after *V. cholerae* MV treatment. **D**. Down-regulated genes after *V. cholerae* MV treatment. Mann–Whitney U test was used to determine statistical differences.

Supplementary Figure 2

A Genes enriched towards GO term: epithelial cell differentiation: RNA expression in HCT8 cells

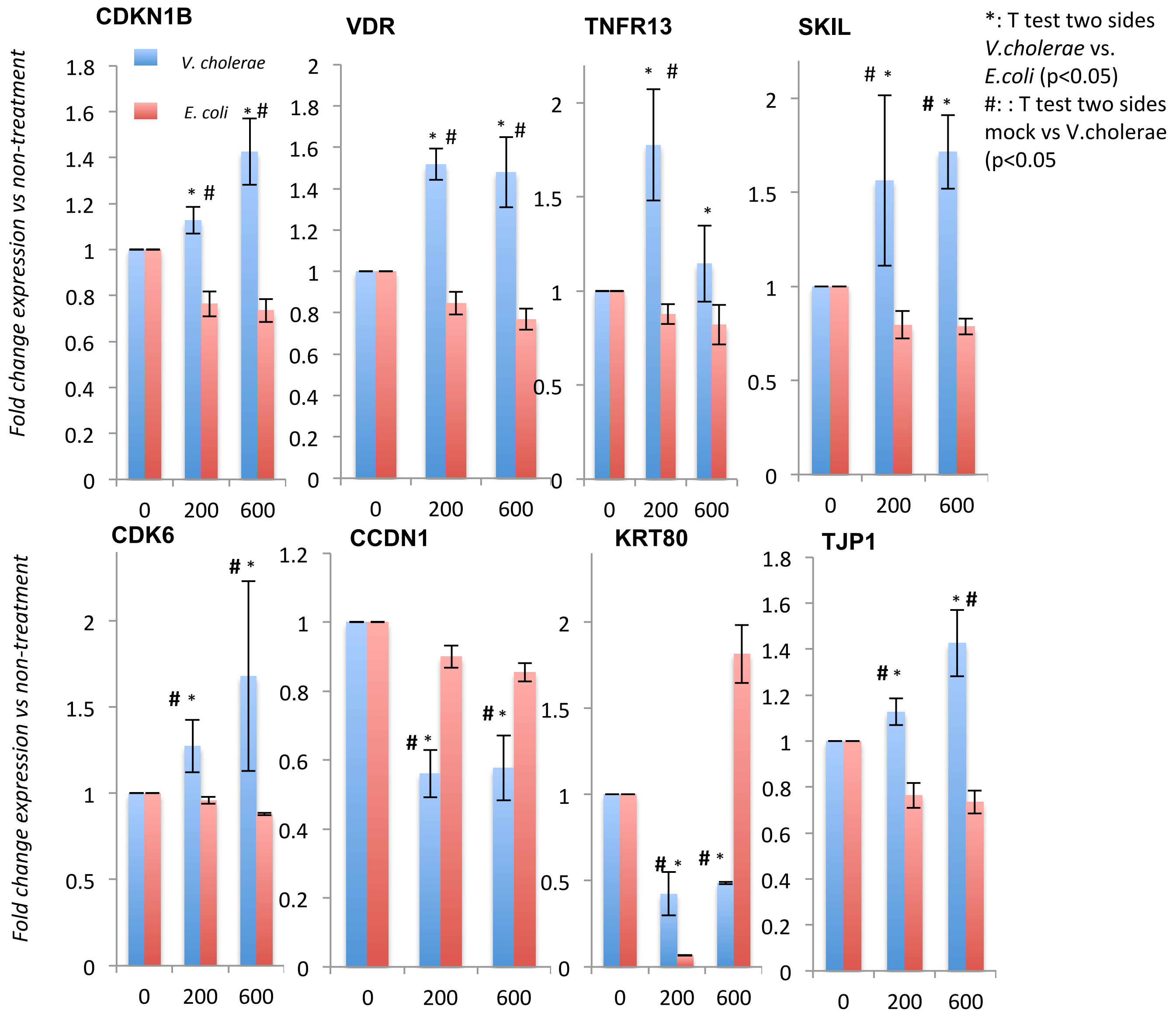

B *V. cholerae* MVs and *E. coli* up-regulated genes: RNA expression in HCT8 cells

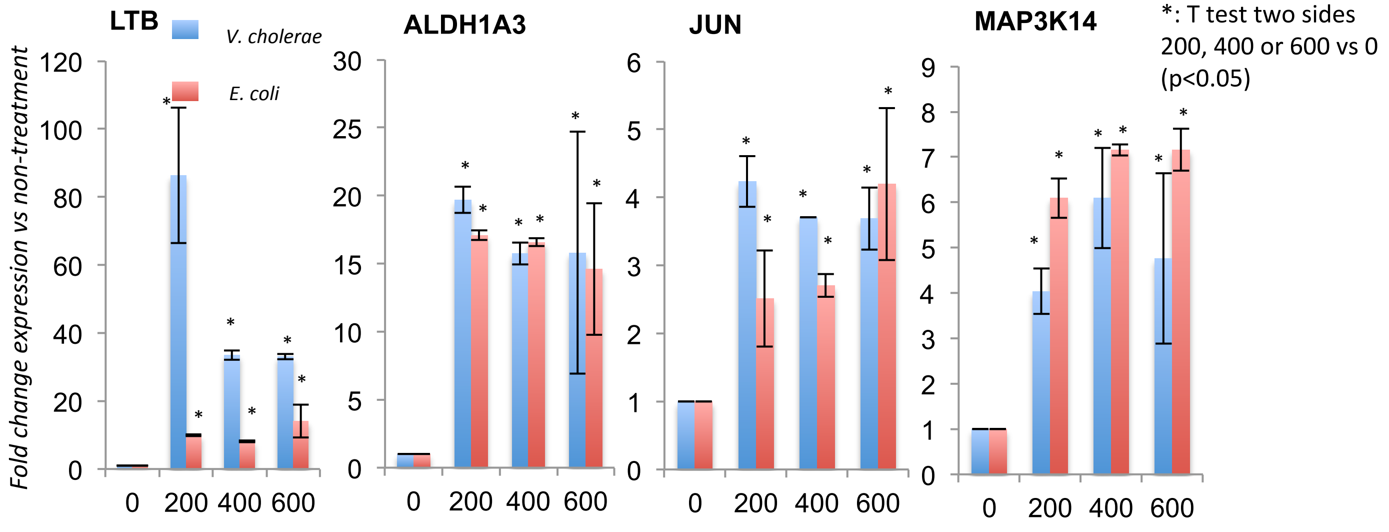

Continuation of Supplementary Figure 2

C Genes enriched towards GO term: epithelial cell differentiation: RNA expression in MCF-7 cells for *Vibrio cholerae* MVs

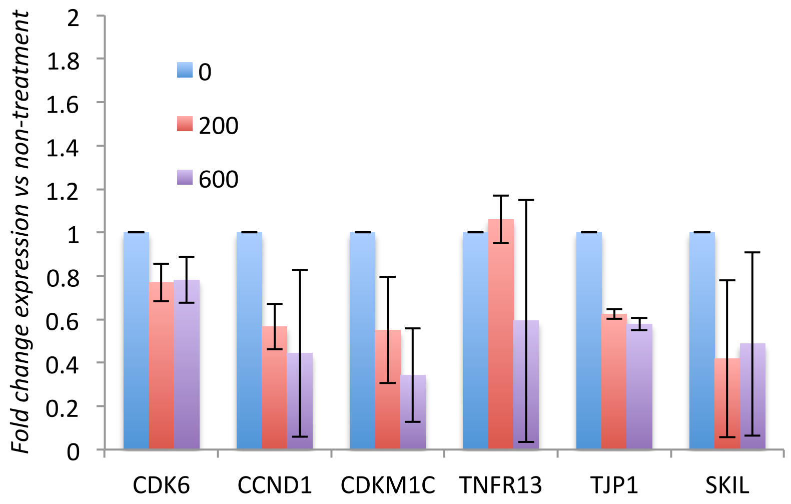

Figure S2. Supplementary information for Figure 1. A. Realtime PCR of gene transcripts enriched towards cell differentiation GO enrichment. The PCR was performed from HCT8 cells co-cultured with different concentrations of MVs from *E. coli* or *V. cholerae* for 5 hours. **B.** Realtime PCR of gene transcripts commonly regulated by MVs from *V. cholerae* or *E. coli*. The PCR was performed from HCT8 cells co-cultured with different concentrations of MVs from *E. coli* or *V. cholerae* for 5 hours. **C.** Realtime PCR of gene transcripts enriched towards cell differentiation GO enrichment. The PCR was performed from MCF-7 cells co-cultured with different concentrations of MVs from *V. cholerae* for 5 hours. The experiments were performed in triplicate and SD is shown.

Supplementary Figure 3

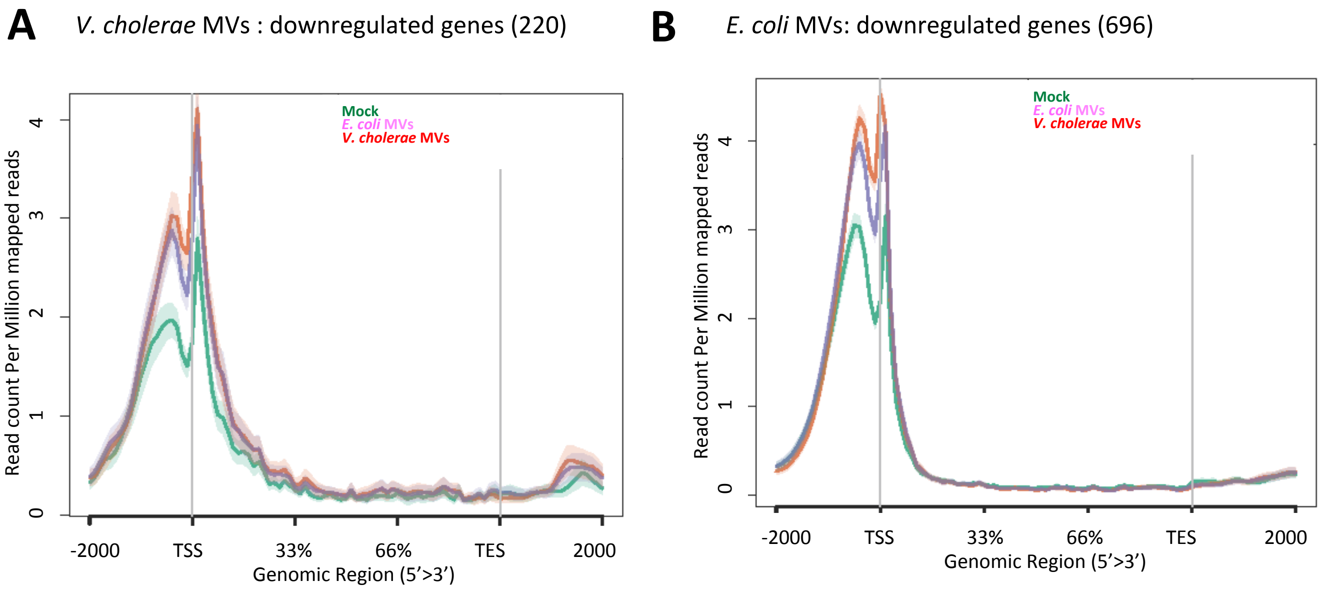

Figure S3. Supplementary information for Figure 2. A. H3K4me3 signal at TSS of down-regulated genes from HCT8 cells co-cultured with MVs from *V. cholerae.* **B.** H3K4me3 signal at TSS of downregulated genes from HCT8 cells co-cultured with MVs from *E. coli.*
